# Physicians’ Visiting List for 1872

**Published:** 1871-12

**Authors:** 


*Physicians' Visiting List for* 1872. Philadelphia, Lindsay & Blakeston.

This is an able and favorite memorandum book, and it seems that there is no occasion to speak of its necessity to physicians. It is made in different styles of binding to suit all wants; the one we have received closing with the tuck as formerly. This is adapted to all the different necessities of physicians, in style, size, arrangement etc. Almost all physicians are familiar with its use and it requires no further description.

